# Correspondence

**Published:** 1864-02

**Authors:** B. Wood


### Correspondence.

Editors of the Register :—I enclose the following letter relating a case of filling upon an exposed nerve with the fusible metal. It is probably the first experiments of the kind actually tried with this filling. It may be that the heat cauterizes the surface of the pulp and is beneficial : and, with permission of the writer, I submit his remarks to the Register by way of inciting observation to this point.

In conversation, on the occasion referred to, I spoke of the former practice of cauterizing the pulp, not only with caustics, but with the actual cautery, with a view to stimulating healthy action; and expressed the opinion that the Plastic Metallic Filling might be applied in such cases with impunity, except the pain, which I took for granted would be severe for the time, not having tried it myself, but which in the cases alluded to proved to be comparatively slight. Mr. S. is a young practitioner and student, has used the Filling only about three months, but has acquired a success with it that would do credit with any operator.

Yours, &c.,

B. WOOD.

